# Exploring the Teleproctoring Potential of Telesurgery: The First Remote Procedures Performed Simultaneously Between Orlando and Shanghai

**DOI:** 10.1590/S1677-5538.IBJU.2025.0083

**Published:** 2025-03-20

**Authors:** Marcio Covas Moschovas, Shady Saikali, Travis Rogers, Mischa Dohler, Michael Mcdonald, Ela Patel, Jeffrey Marquinez, Ahmed Gamal, Jeffery Magnuson, Vipul Patel

**Affiliations:** 1 AdventHealth Global Robotics Institute Kissimmee FL USA AdventHealth Global Robotics Institute, Kissimmee, FL, USA; 2 University of Central Florida Orlando FL USA University of Central Florida - UCF, Orlando, FL, USA; 3 Advanced Technology Group, Ericsson Inc. Santa Clara USA Advanced Technology Group, Ericsson Inc., Santa Clara, USA

**Keywords:** Robotic Surgical Procedures, Minimally Invasive Surgical Procedures, Prospective Studies

## Abstract

**Introduction:**

We performed the first study exploring telesurgery's teleproctoring potential while performing long-distance procedures between Orlando (USA) and Shanghai (China) over a distance of 13,000 km. The objective was to evaluate telesurgery's performance and teaching potential using the MicroPort® MedBot™ robotic platform and fiber-optic technology in real-time collaboration during urologic procedures.

**Materials and Methods:**

We simulated a real-life scenario where surgeons could communicate and send mutual inputs during telesurgery cases. A prospective study using live porcine models was conducted on July 23–24, 2024. Surgeons in Orlando and Shanghai took turns controlling the robotic system, performing nephrectomies, pyeloplasties, and ureteroureterostomies while transferring control between locations. Latency and system performance were continuously monitored, and real-time communication between the surgeons was facilitated by fiber-optic technology.

**Results:**

Surgeons successfully completed numerous urologic procedures, including nephrectomies, pyeloplasties, and ureteroureterostomies, with seamless control transfers. Remote surgeons provided teleproctoring and assistance during the procedures. The robotic system operated without issues throughout the two-day study. The median latency was 139 milliseconds (range 137–216 ms) on the first day and 139 milliseconds (range 137–185 ms) on the second day.

**Conclusions:**

This study demonstrates the feasibility of long-distance telesurgery and highlights its potential to improve surgical outcomes, facilitate training, and offer remote assistance for complex cases. Telesurgery could play a significant role in expanding access to specialized care and enhancing robotic surgical training globally.

## INTRODUCTION

Telesurgery, the practice of performing surgical procedures remotely using robotic technology and advanced telecommunications, represents one of the most transformative advancements in modern medicine (1-3). By combining the precision of robotics with the connectivity of telecommunication networks, telesurgery has the potential to overcome geographical barriers, making specialized surgical care accessible to patients in underserved and remote areas (4, 5). Since the beginning, this innovative approach has sparked significant interest within the medical community in addressing disparities in healthcare delivery and optimizing patient outcomes.

This surgical approach began with the historic Lindbergh operation in 2001, where a surgeon in New York performed a successful gallbladder removal on a patient in France, over 6,000 kilometers away (6). This landmark achievement demonstrated the feasibility of remote surgical interventions and highlighted the potential of telesurgery to revolutionize the delivery of care. However, implementations in the following years faced numerous challenges, including limited connectivity, latency in data transmission, high costs, and the need for advanced robotic platforms. Despite these limitations, rapid advancements in technology, such as high-speed 5G networks, fiber-optic connectivity, and next-generation robotic systems, have gradually overcome these obstacles, bringing telesurgery closer to widespread clinical application (7-11).

Beyond its potential to provide direct patient care, telesurgery offers immense potential for advancing and changing the history of surgical education and training. By enabling expert surgeons to guide and mentor trainees in real-time across vast distances, telesurgery can expand access to high-quality training in robotic techniques, particularly in low-resource settings. While these developments signal a promising future for telesurgery, its adoption remains in its early stages, with significant ethical, technical, and legal considerations requiring further exploration (12-14). After performing a study evaluating the feasibility of remote surgery over long distances (15), we conducted a pioneer Telesurgery study to simulate a real-life scenario where surgeons on the opposite side of the connection could control the robot and send inputs during the cases as Teleproctors. Using live porcine models, we evaluated the connection and robotic performances between Orlando (USA) and Shanghai (China) using surgeons in both centers (13,000km apart) operating simultaneously on the same animals (with telesurgery consoles on both sides).

## MATERIALS AND METHODS

On July 23rd and 24th, 2024, we conducted a prospective telesurgery study using live animal models (porcine) connecting one robotic training center in Orlando (USA) to another training center in Shanghai (China). Four surgeons (VP, SS, TR, MM) were in Orlando (Nicholson Center) and one (MCM) in Shanghai (MicroPort® animal lab). Our hypothesis is that telesurgery can be used as a novel tool for teaching robotic surgery across the globe while minimizing complications due to its potential as a teleproctor and the real-time interaction between surgeons from different centers. The endpoint of the study was to evaluate the long-distance connectivity among distant countries and the surgical performance while the surgeons (13,000km apart) were operating on the same animals and switching the command of the consoles. In this context, we could simulate a real teleproctoring scenario using telesurgery technology with a remote surgeon taking the procedure over in cases of complications or challenging scenarios. This is the first study in the literature that is designed, and we wanted to address the potential of historical changes in the way that robotic surgery is trained and delivered.

The secondary endpoint was the comparison of the technical parameters of the connection, including speed, delay, and frame loss, when transmitting data from Shanghai to Orlando and vice versa. We wanted to identify any directional differences in signal quality and their potential impact on surgical performance. None of the studies in the literature ever reported if we have differences in the signal performance when it is transported from China to the USA and back.

### Animal study

Prior to the study, the research project received approval from the Experimental Animal Ethics Committee at Timely Horse (Shanghai) Laboratory Animal Equipment Co., LTD, Shanghai (China), and from the Nicholson Center (Celebration, FL, USA). All procedures were conducted in strict compliance with the institutional animal care ethical standards and regulatory requirements of both centers, following the Institutional Animal Care and Use Committee (IACUC) under the protocol identified as #MedBot2024, approved on July 18th, 2024 (16). Additionally, our study followed the EQUATOR and ARRIVE reporting guidelines for research involving animal models (17).

We conducted the study over two days, using four animals. Each day, we began the procedures with one animal in Shanghai at 7 PM local time (7 AM in Orlando). After completing the surgery in Shanghai, we immediately proceeded with the operation on the animal in Orlando. Throughout the trial, the surgeons were able to switch roles and take over the cases multiple times, demonstrating the flexibility and collaborative potential of the system during telesurgery procedures.

### Robotic Platform and network connection

We used the MicroPort® MedBot™ robotic platform (Shanghai MicroPort MedBot Group Co., Ltd.), featuring an immersive closed console and four robotic arms attached to a single patient-side cart (18). The platform has three 8mm instrument trocars and one 11mm trocar for the 10mm scope, with both 0 and 30-degree scopes available. In our study, we used scissors, Cadiere, and bipolar instruments. The 30-degree scope angle can be adjusted via the console's touchpad and the control of the instruments can be switched among the surgeons using the touchpad as well (Figure-2).

**Figure 1:**
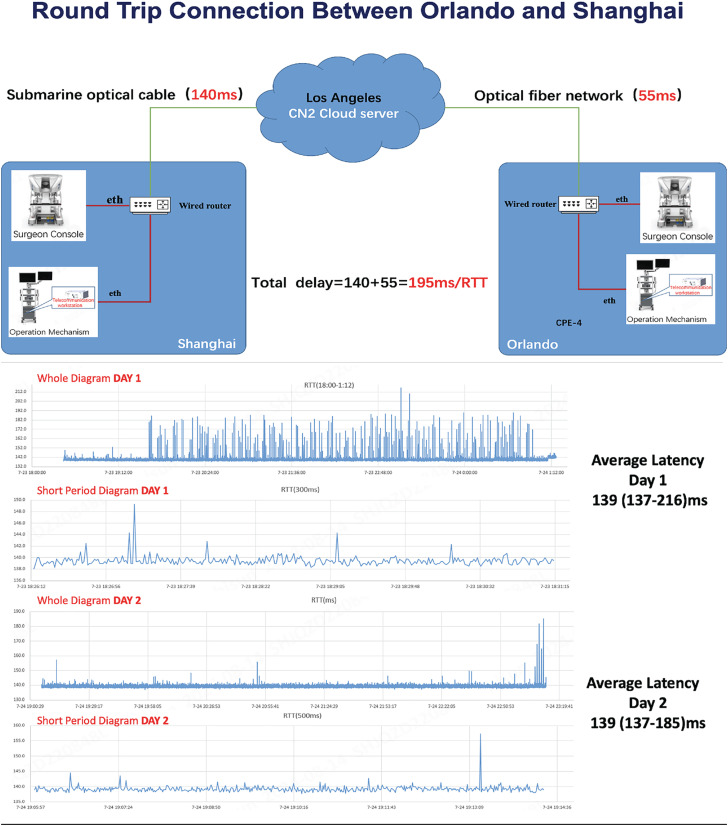
Roundtrip connection details between Orlando and Shanghai. Total delay displayed in milliseconds (ms). Average latency in milliseconds displayed in graphs during the whole procedure.

**Figure 2:**
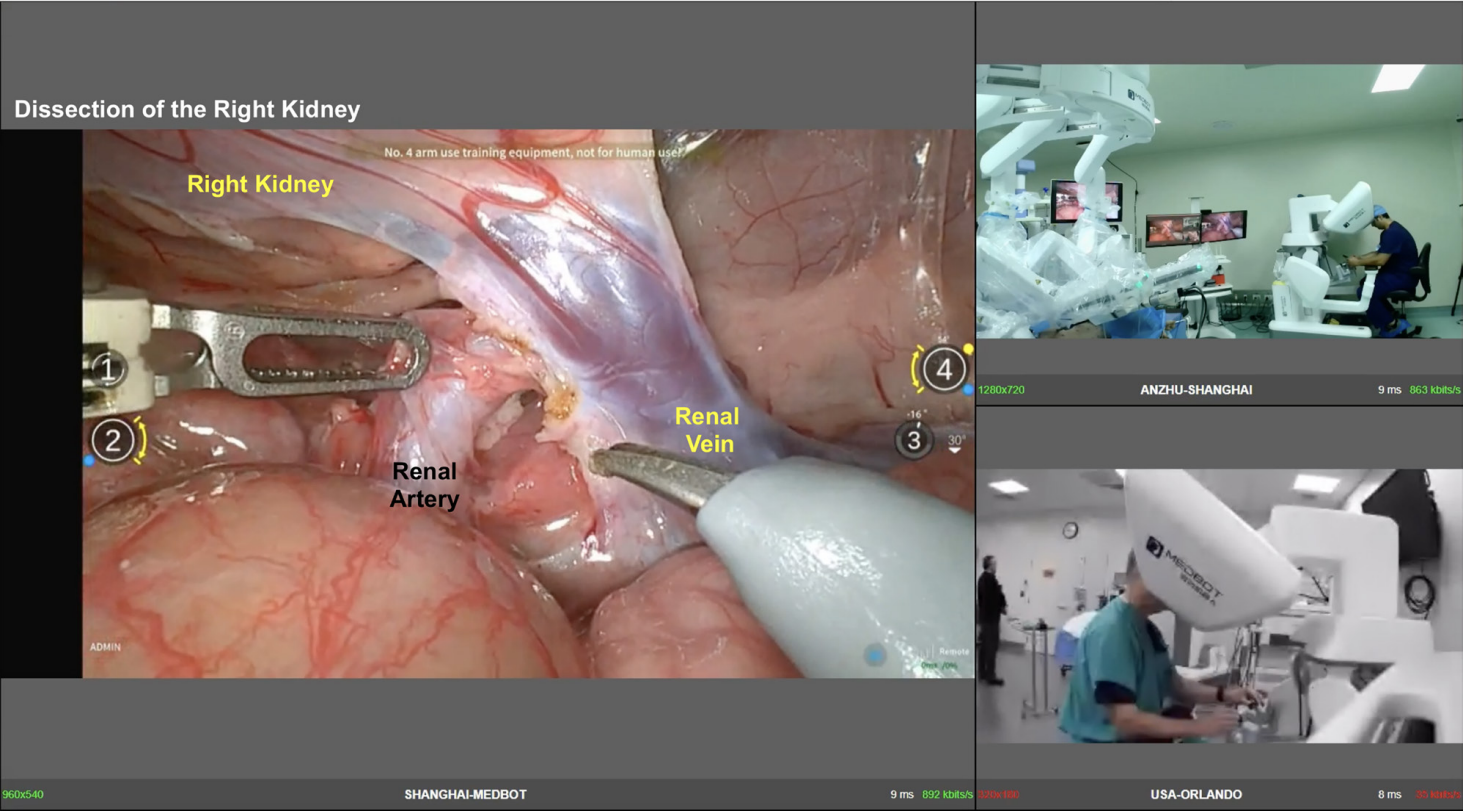
Illustration of the interaction of one surgeon in Shanghai (China) and another in Orlando (USA). We illustrated a dissection of the right renal hilum on the left side screen with the kidney on the top and the renal artery and vein on the center. This figure illustrates the same interface the surgeons have in their room while performing the cases. During the case, with an accessory screen, it is possible to watch the surgery, the remote surgeon, and your own image.

We used corporate Wi-Fi (Fiber) combined with continental and transpacific fiber, as shown in Figure-1. The data travels from the console in Shanghai to a wired router until reaching a submarine optical cable. In sequence, the signal reaches the cloud server in Los Angeles (USA) and travels through an optical fiber until reaching another wired router in Orlando connected to the console.

### Surgeries Performed

Several urologic procedures were performed during the two days of trial, including 8 radical nephrectomies, 8 partial nephrectomies, 2 ureteroureterostomies, and 2 pyeloplasties (Table-1). The animals underwent general anesthesia and monitoring. In sequence, they were positioned in lateral decubitus and the robotic trocars were placed in the paramedian line with one 12mm assistant trocar between the first and second (Scope) proximal trocars. After the procedures, the animals were euthanized by the lab with appropriate technique, following the ethical standards of animal management in surgical training and animal studies.

**Table 1:** Illustration of all surgeries performed in four animals during the two-day trial. Latency was described in milliseconds with mean value and range for both days. Total operative time was described in minutes from the trocar placement until the end of the procedure.

Procedure Type	Animal Model	Surgeon Location	Animal Location	Connection Used	Platform Used	Latency (Milliseconds)	Complications	Troubleshooting with the Robot	Total Operativetime (Minutes)
2 partial nephrectomies 2 radical nephrectomies 2 ureteroureterostomies 1 pyeloplasty	Porcine	Orlando (USA)	Shanghai (China)	Fiber	Microport Medbot Toumai	139 (137-216)	no	no	180 minutes
2 partial nephrectomies 2 radical nephrectomies	Porcine	Shanghai (China)	Orlando (USA)	Fiber	Microport Medbot Toumai	139 (137-216)	no	no	150 minutes
2 partial nephrectomies 2 radical nephrectomies	Porcine	Shanghai (China)	Orlando (USA)	Fiber	Microport Medbot Toumai	139 (137-185)	no	no	140 minutes
2 partial nephrectomies 2 radical nephrectomies 1 ureteroureterostomy 1 pyeloplasty	Porcine	Orlando (USA)	Shanghai (China)	Fiber	Microport Medbot Toumai	139 (137-185)	no	no	170 minutes

## RESULTS

Figure-1 presents the latency data for the telesurgery connection between Orlando and Shanghai, detailing the signal transmission path. The process begins at the surgeon's console, which sends control signals to the telecommunication workstation. From there, the data is relayed through a wired router and transmitted via a submarine optical cable to a cloud server located in Los Angeles (LA). Once the signal reaches LA, it is routed through a high-speed terrestrial fiber-optic network, ensuring minimal latency as it travels to its final destination in Orlando. This infrastructure enables real-time communication, which is critical for the precision and responsiveness required in telesurgical procedures.

On the first day of testing, the mean latency was recorded at 139 milliseconds, with a range of 137 to 216 milliseconds. On the second day, the mean latency remained consistent at 139 milliseconds, though with a slightly reduced range of 137 to 185 milliseconds. We can visualize the connection pattern illustrated in the graphs in both days. A comparative analysis of both transmission directions—Orlando to Shanghai and Shanghai to Orlando—revealed no significant differences in speed, latency, or frame loss, even after several hours of continuous robotic operation. Throughout the trial, no technical complications or robotic malfunctions were observed, and no troubleshooting was required. Overall, the telesurgery connection remained stable and functioned effectively, demonstrating its reliability for remote surgical procedures.

Detailed operative data is provided in Table-1. On the first animal (in Orlando) we performed 1 pyeloplasty, 2 ureteroureterostomies, 2 partial nephrectomies, and 2 radical nephrectomies. On the second animal (in Shanghai), we performed 2 partial nephrectomies, and 2 radical nephrectomies. On the third animal (in Shanghai) we performed 2 partial nephrectomies, and 2 radical nephrectomies. Finally, on the third animal (in Orlando), we performed 1 pyeloplasty, 2 ureteroureterostomies, 2 partial nephrectomies, and 2 radical nephrectomies.

## DISCUSSION

After performing several studies and having experience with several telesurgical platforms worldwide(15, 19-21), we designed a two-day study reproducing a real-life telesurgery scenario where both ends of the connection have control over the robot, showing the teleproctoring potential of remote surgery. We believe remote surgery is the next frontier to perform historical changes in how robotic surgery is trained and delivered. In our project, connectivity and robotic technology performed optimally on both ends. Latency remained within an acceptable range, allowing for smooth, complication-free procedures. The robotic system operated clearly for several hours without any troubleshooting or malfunctions. A notable aspect of the trial was the ability to switch the console control multiple times during surgeries. Surgeons, positioned 13,000 km apart, could communicate, discuss cases in real-time, and transfer control in key steps of the surgery in real-time when needed. This simulation underscores the realistic teleproctoring potential of telesurgery, particularly when an experienced remote surgeon steps in to assist another surgeon during complex or challenging procedures (19, 22). It highlights Telesurgery's potential for improving robotic surgery training while optimizing surgical outcomes.

Teleproctoring allows expert surgeons to guide and supervise less-experienced practitioners remotely, ensuring the safe and effective adoption of advanced surgical techniques. This capability is particularly crucial for institutions in remote or underserved regions where access to specialized training is limited (23, 24). Through telesurgery platforms, proctors can observe live surgical procedures, provide real-time feedback, and even take control of the robotic console if necessary, offering a level of oversight comparable to in-person mentorship. By enabling this transfer of knowledge, teleproctoring provides the development of local surgical expertise and enhances patient outcomes in regions where high-level surgical skills are scarce. In our study, different surgeons with diverse levels of expertise could discuss in real time the best options for that specific step of the surgery. In some moments, the remote surgeon interfered and assumed control of the machine to show the best way of performing that step, and in other moments, he only sent opinions as a teleproctor.

In the context of robotic surgery training, telesurgery opens new avenues for skill development and global collaboration. Traditionally, surgeons must travel to specialized centers to receive hands-on training under experienced mentors, a process that can be logistically and financially challenging. Telesurgery eliminates these barriers by allowing trainees to practice and refine their skills under the remote supervision of experts. Using immersive technologies such as high-definition imaging and virtual reality integration, remote mentors can provide precise guidance, ensuring that trainees achieve the necessary competencies. This democratization of access to surgical training is particularly relevant for emerging healthcare markets, where building local capacity in robotic surgery can significantly enhance the quality of care. Despite the initial investment in telerobotic technology, this teleproctoring approach could save a lot of resources if we consider the training of several surgeons from multiple specialties who would have to travel and spend time away from their centers to learn robotics in other cities, states, or countries.

Moreover, telesurgery-based training provides an unprecedented opportunity to standardize surgical education globally. By leveraging robotic platforms and telecommunication technologies, institutions can design structured training programs that are accessible to surgeons worldwide. This approach ensures consistent teaching methods, rigorous assessment of skills, and adherence to evidence-based guidelines, regardless of geographic location. The expansion of telesurgery training also supports the rapid dissemination of new techniques and innovations, accelerating their adoption in clinical practice. By integrating teleproctoring and remote mentorship into the broader framework of surgical education, telesurgery has the potential to redefine how surgeons are trained, fostering a more equitable and interconnected global healthcare system. In other words, the same expert could train several surgeons worldwide on the same day, which enables several international scientific collaborations with standardized surgical techniques and prospective multicentric studies performed on that specific surgical approach. This would improve the quality of the current literature with a higher level of evidence.

Our study demonstrates real-life scenarios where the teleproctor in Shanghai assumes control of the robotic console during critical phases of the procedure. These examples highlight the potential of remote teleproctoring to enhance surgical precision by providing guidance for the identification and dissection of correct anatomical planes, reducing complications, and optimizing patient outcomes. Notably, in centers with moderate expertise in robotic surgery, the teleproctor's involvement may be limited to high-risk moments, such as arterial or venous dissections or releasing invasive tumors. However, in facilities with minimal robotic surgery experience, the teleproctor might need to remain connected throughout the entire procedure to ensure safety and efficacy. This differentiation is significant because it directly impacts factors like reimbursement models and medical fees. In cases where the teleproctor's role is limited to a few minutes, the associated costs are minimal compared to potential travel, hotel stays, transportation, and in-person fees. Conversely, scenarios requiring continuous teleproctoring support for several hours demand greater resources and may increase the costs. These distinctions underline the importance of evaluating teleproctoring scenarios to develop standardized guidelines and recommendations for reimbursement policies that consider varying levels of surgical complexity and expertise.

While the potential benefits of teleproctoring via telesurgery technology are evident, the field currently lacks comprehensive guidelines and robust literature on credentialing, training, and certification. Standardized evaluation using teleproctoring could pave the way for remote certification programs, facilitating the establishment of globally recognized credentials that ensure consistency and reliability in assessing surgical skills. This technology holds immense promises for verifying a trainee's competency in specific robotic procedures, creating a structured pathway for remote credentialing in robotic surgery. Therefore, it is vital for surgical societies with expertise in robotic surgery and telesurgery to take an active role in developing guidelines and recommendations about remote surgery certification and training. Their involvement would ensure the safe, ethical, and effective implementation of remote surgery practices on a global scale (22). By addressing these gaps in standardization and governance, teleproctoring can evolve into a transformative tool for training and credentialing remote surgeons, ultimately advancing the field of robotic surgery worldwide.

Progress and improvements have been noted in the last decades; however, connectivity still remains a potential challenge in telesurgery procedures because its optimal performance depends on several factors (12). The first one is distance (exact routing path between both endpoints) because the signal speed inside the fiber is around 200.000 km/s, and the roundtrip of our study was approximately 26.000km. Therefore, the minimum delay allowed by physics would be 130ms. In this scenario, we connected one robot on each side of the line to evaluate the connection and robot performance on the two ways of transmission. Consequently, we consider that our connection was of high quality on both days because considering the distance and all paths taken to reach both centers, in some periods of the trial, we reached less than 140 milliseconds. A second source of latency is network congestion, such as fiber networking congestion during peak hours. A third source of networking latency is the wireless connection from the mobile device to the access point if an untethered design is chosen. In non-congested situations and with sufficient strength of connection, the Wi-Fi air interface adds another 3ms latency, and the 5G air interface adds around 10ms (1).

Despite the pioneering and innovative aspects of our study, limitations must be acknowledged because the results from animal studies may not fully reflect human outcomes due to species-specific variations in anatomy, physiology, and treatment responses. However, we are optimistic about the results of this pioneering study, and we believe this project provides strong proof of concept that teleproctoring using long-distance telesurgery among two cities or countries is indeed feasible and surgeons can have real-time inputs from experts to improve surgical outcomes and potentially improving robotic surgery certification worldwide.

## CONCLUSION

This study represents the first demonstration of teleproctoring applications using telesurgery technology in a preclinical setting, bridging the 13,000-kilometer distance between Orlando and Shanghai. Achieving and maintaining optimal, stable network connectivity was crucial to the success of these procedures. Surgeons switched robotic control during operations without complications or delays, showing the reliability and efficiency of this technology. Our findings highlight the immense potential of telesurgery to facilitate real-time communication and collaboration between surgeons across vast distances. By enabling the exchange of expertise and guidance during critical moments, telesurgery has the capacity to minimize complications, enhance surgical outcomes, and improve robotic surgery training. This work serves as proof of concept for long-distance remote surgery, setting the stage for further clinical trials. As the field advances, studies like this will be instrumental in overcoming the remaining challenges and fully realizing telesurgery's humanitarian and clinical potential to transform global healthcare delivery.
